# Xanthine Derivatives Reveal an Allosteric Binding Site in Methylenetetrahydrofolate Dehydrogenase 2 (MTHFD2)

**DOI:** 10.1021/acs.jmedchem.1c00663

**Published:** 2021-08-02

**Authors:** Lung-Chun Lee, Yi-Hui Peng, Hsin-Huei Chang, Tsu Hsu, Cheng-Tai Lu, Chih-Hsiang Huang, Ching-Cheng Hsueh, Fang-Chun Kung, Ching-Chuan Kuo, Weir-Torn Jiaang, Su-Ying Wu

**Affiliations:** † Institute of Biotechnology and Pharmaceutical Research, National Health Research Institutes, 35, Keyan Road, Zhunan Town, Miaoli County 350 Taiwan, ROC; ‡ Graduate Institute of Biomedical Sciences, China Medical University, Taichung 406, Taiwan, ROC

## Abstract

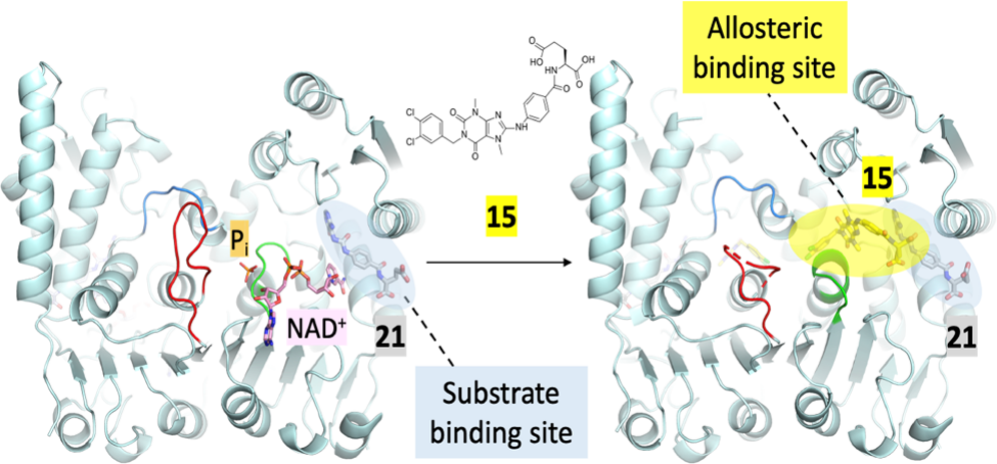

Methylenetetrahydrofolate dehydrogenase 2 (MTHFD2) plays an important role in one-carbon metabolism. The MTHFD2 gene is upregulated in various cancers but very low or undetectable in normal proliferating cells, and therefore a potential target for cancer treatment. In this study, we present the structure of MTHFD2 in complex with xanthine derivative **15**, which allosterically binds to MTHFD2 and coexists with the substrate analogue. A kinetic study demonstrated the uncompetitive inhibition of MTHFD2 by **15**. Allosteric inhibitors often provide good selectivity and, indeed, xanthine derivatives are highly selective for MTHFD2. Moreover, several conformational changes were observed upon the binding of **15**, which impeded the binding of the cofactor and phosphate to MTHFD2. To the best of our knowledge, this is the first study to identify allosteric inhibitors targeting the MTHFD family and our results would provide insights on the inhibition mechanism of MTHFD proteins and the development of novel inhibitors.

## Introduction

Cellular metabolism is substantially altered during tumorigenesis and malignant progression. One-carbon metabolism produces one-carbon units for various cellular processes and hyperactivation of this pathway drives oncogenesis.^1^

MTHFD2, a one-carbon metabolism enzyme, is localized in the mitochondria and plays an important role in nucleic acid biosynthesis, amino acid metabolism, and protein synthesis.^2,3^ It is a NAD-dependent enzyme and functions as both 5,10-methylenetetrahydrofolate (CH_2_-THF) dehydrogenase and 5,10-methenyltetrahydrofolate (CH^+^-THF) cyclohydrolase, converting CH_2_-THF to 10-formyl-THF (CHO-THF).^4,5^ Recently, studies have revealed that MTHFD2 is a highly expressed metabolic gene in human cancer based on a comprehensive analysis of the RNA profiles of 1454 metabolic enzymes across 1981 tumors spanning 19 cancer types.^6^ In addition, elevated MTHFD2 expression is associated with poor prognosis in both hematological and solid malignancies.^6−8^ Although MTHFD2 is upregulated in various cancers, transformed cells, and developing embryos, it is present only in very low or undetectable levels in normal postmitotic and proliferating cells. Moreover, depletion of MTHFD2 impairs aggressive phenotypes and causes cell death in multiple cancers, including breast cancer, colorectal cancer, central nervous system tumors, lung cancer, ovarian cancer, renal cancer, melanoma, and leukemia.^6−8^ These results suggested that targeting MTHFD2 is a promising strategy for cancer therapy.^9,10^

MTHFD1 is a homologous protein of MTHFD2^11,12^ whose dehydrogenase/cyclohydrolase domain shows 55.6% sequence similarity with MTHFD2 as analyzed by the Needle program in the European Molecular Biology Open Software Suite (EMBOSS).^13^ MTHFD1 performs the same reactions as observed in MTHFD2 but is located in the cytoplasm instead of the mitochondrion. The most distinct profile of these two proteins is MTHFD1 that is expressed in normal cells and tissue but MTHFD2 is absent in normal cells. Therefore, the design of inhibitors selectively targeting MTHFD2 is anticipated to offer a larger therapeutic window with reduced toxicity and side effects.

**Figure 1:**
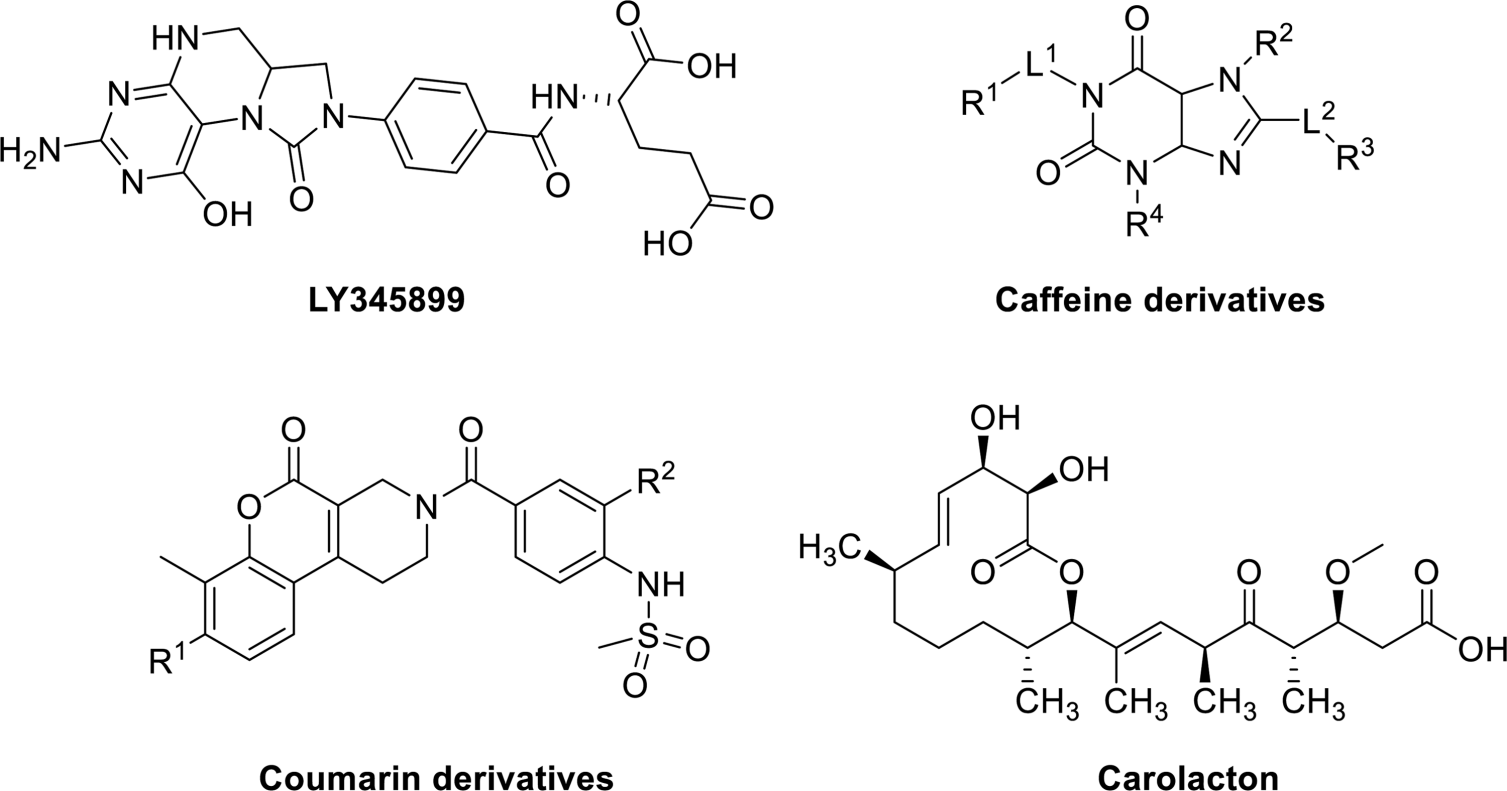
Reported MTHFD2 inhibitors.

Some MTHFD2 inhibitors have been reported including LY345899 (Figure 1), a folate analogue developed by Eli Lilly, which enzymatically inhibited MTHFD2 with an IC_50_ value of 663 nM. LY345899 exhibited potent antitumor activity in colorectal cancer in vivo^7^ but was poorly selective among MTHFD isozymes and an even more potent inhibitor of MTHFD1 (IC_50_ = 96 nM).^14^ Raze Therapeutics reported a new class of MTHFD2 inhibitors based on a caffeine scaffold (Figure 1).^15,16^ Kawai et al. disclosed a series of sulfonamide derivatives incorporating a coumarin skeleton (Figure 1). They were selective MTHFD2 inhibitors^17,18^ and showed antitumor efficacy in a mouse xenograft model.^18^ Moreover, Fu et al. reported that the natural product carolacton (Figure 1) occupied the substrate pocket of the MTHFD ortholog, bacterial FolD, and showed inhibition toward both human MTHFD1 and MTHFD2.^19^

Although several structurally diverse MTHFD2 inhibitors have been reported, only a few crystal structures of MTHFD2 bound to an inhibitor have been disclosed. One example is that of human MTHFD2 in complex with LY345899, NAD^+^, and phosphate.^14^ MTHFD2 forms a homodimer and each monomer consists of the N-lobe and C-lobe connected by two long α helices. LY345899 was found to bind MTHFD2 in the substrate-binding site, a large cleft between the two lobes. The pteridine moiety of LY345899 formed extensive interactions with the protein and the glutamyl tail extended into the solvent region. While the cofactor NAD^+^ bound to the C-lobe near the substrate-binding site and was stabilized by the classical dinucleotide-binding Rossmann motif in MTHFD2, the phosphate interacted with the hydroxyl group of NAD^+^ and formed hydrogen-bond interactions with residues in the dimer interface. Recently, Kawai et al. reported the structures of human MTHFD2 in complex with tricyclic coumarin derivatives.^17,18^ Coumarin derivatives also occupied the substrate-binding site but adopted a slightly different binding mode compared to LY345899. Instead of establishing a hydrogen-bond network as observed in the pteridine moiety of LY345899, the interactions of the coumarin scaffold and the terminal moiety with the surrounding residues contributed to the inhibition of MTHFD2 by these compounds.

In this study, we present the structure of MTHFD2 in complex with xanthine derivative **15**. **15** was found to occupy a novel binding site entirely different from that occupied by the canonical substrates and the cofactor. It allosterically bound to MTHFD2 and coexists with the substrate analogue. Even more interestingly, MTHFD2 was found to undergo several conformational changes upon the binding of **15** that subsequently obstructed the binding of cofactor NAD^+^ and phosphate to MTHFD2. Furthermore, structure–activity relationships, kinetic studies, and several crystal structures of MTHFD2 complexed with xanthine derivatives are also presented to elucidate the mechanism of inhibition of MTHFD2 by this series of compounds.

## Results

### Chemistry

**Scheme 1:**
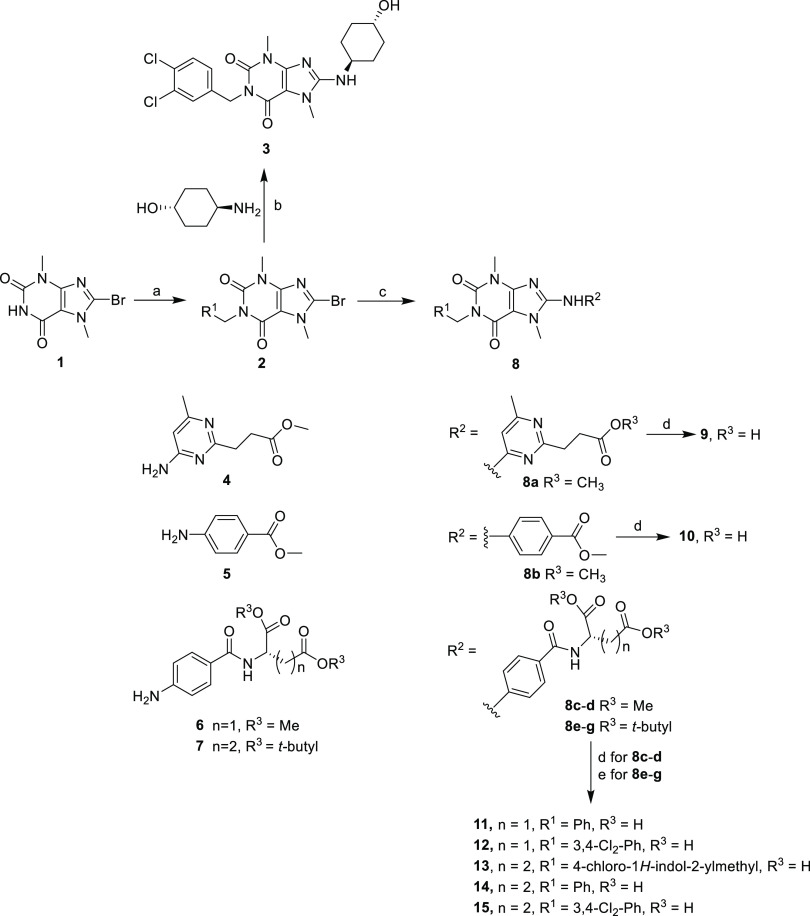
Reagents and Conditions (a) PhCH_2_Br, 3.4-Cl_2_-PhCH_2_Br or 4-chloro-2-chloromethyl-1*H*-indole, K_2_CO_3_, DMF, 50 °C, 8 h, 72–79%; (b) *trans*-4-aminocyclohexanol HCl, DIPEA, 1-butanol, 120 °C, 72 h, 41%; (c) aromatic amine **4**, **5**, **6**, or **7**, Pd(OAc)_2_, xantphos, Cs_2_CO_3_, DMF, 120 °C, 1 h, 31–42%; (d) 2 N NaOH, DMF, 12 h, 50%; and (e) TFA, 25 °C, 0.5 h, 70–74%.

3,7-Dimethyl-3,7-dihydropurine-2,6-diones (xanthine derivatives) **3** and **9**–**15** were prepared, as previously described^16^ (Scheme 1). *N*1-Alkylation of the commercially available 8-bromo-3,7-dimethyl-3,7-dihydro-1*H*-purine-2,6-dione **1** with an appropriate alkyl halide in the presence of Cs_2_CO_3_ in DMF at 50 °C gave intermediate **2**. Nucleophilic substitution of **2** with *trans*-4-aminocyclohexanol using DIPEA in 1-butanol at 120 °C yielded 8-alkylamine substituted **3**. Buchwald–Hartwig coupling of **2** with the aromatic amines **4**, **5**, **6**, or **7** catalyzed by Pd(OAc)_2_ in the presence of xantphos and Cs_2_CO_3_ provided intermediate **8**. Hydrolysis of the methyl ester group (**8a**–**d**) by NaOH in aqueous DMF or deprotection of the *tert-*butyl ester group (**e**–**g**) by trifluoroacetic acid (TFA) yielded the 8-aromatic amine-substituted acids **9**–**12** and acids **13**–**15**, respectively.

**Scheme 2:**
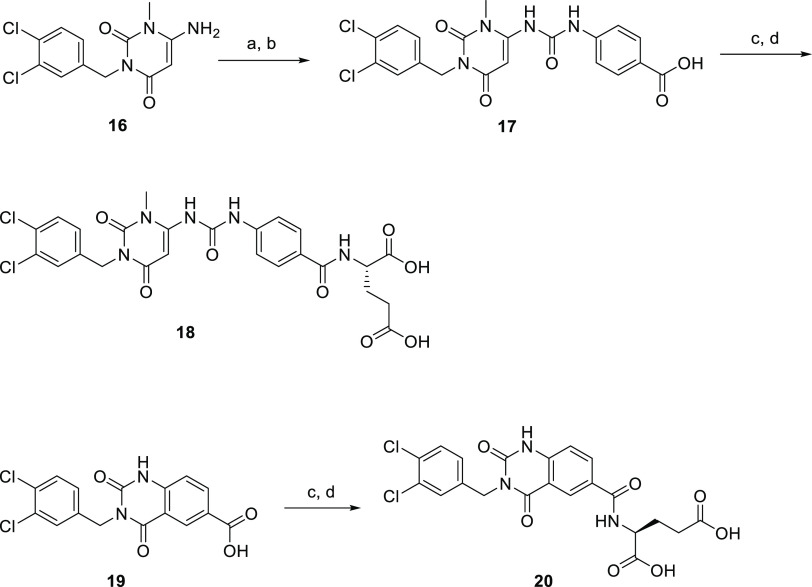
Reagents and Conditions (a) Ethyl 4-isocyanatobenzoate, TEA, THF, room temp., 3 h, 42%; (b) 1 N NaOH, THF, 60 °C, 0.5 h, 63%; (c) l-glutamic acid di-*tert*-butyl ester hydrochloride, HATU, TEA, DMF, 25 °C, 8 h, 58%; and (d) TFA, 25 °C, 0.5 h, 75%.

1-Methyl-1*H*-pyrimidine-2,4-diones **17** and **18** and 1*H*-quinazoline-2,4-dione **20** were synthesized, as depicted in Scheme 2. The 6-amino group-substituted 3-(3,4-dichlorobenzyl)-1-methyl-1*H*-pyrimidine-2,4-dione **16**(20) and 6-carboxyl group-substituted 3-(3,4-dichlorobenzyl)-2,4-dioxo-1,2,3,4-tetrahydroquinazoline **19**(21) were prepared, as previously described. Compound **16** was coupled with ethyl 4-isocyanatobenzoate and then hydrolyzed using NaOH in THF to yield acid **17**. The carboxyl group of **17** was activated by TATU and subsequent *N*-acylation with l-glutamic acid *tert*-butyl ester to afford ester **18**, which was deprotected using TFA to afford pyrimidine-2,4-dione derivative **18**. The synthetic route for the preparation of the final product quinazoline-2,4-dione derivative **20** is similar to that of **18** (Scheme 2).

### Structure–Activity Relationship of Xanthine Derivatives

The inhibitory activity of xanthine derivatives against MTHFD2 and MTHFD1 are shown in Table 1. Compounds **3** and **9**,^16^ the structures of which were disclosed in the patent published by Raze Therapeutics Inc., inhibited MTHFD2 with IC_50_ values of 4.0 and 0.69 μM, respectively. Structural modifications of these xanthine derivatives were conducted to explore the structure–activity relationship (SAR). The scaffold-based molecular design^22−24^ approach was used for lead optimization. Previous biochemical, enzymatic, and structural studies revealed that the glutamic acid moiety of compounds played an important role in the inhibition of the MTHFD family and other enzymes in the one-carbon metabolism pathway.^14,25,26^ Therefore, a glutamic acid moiety was added to the xanthine core and their inhibitory effects were evaluated. In addition, an aspartic acid moiety had a structure similar to glutamic acid and was also added to the xanthine core to explore SAR.

**Table 1:**
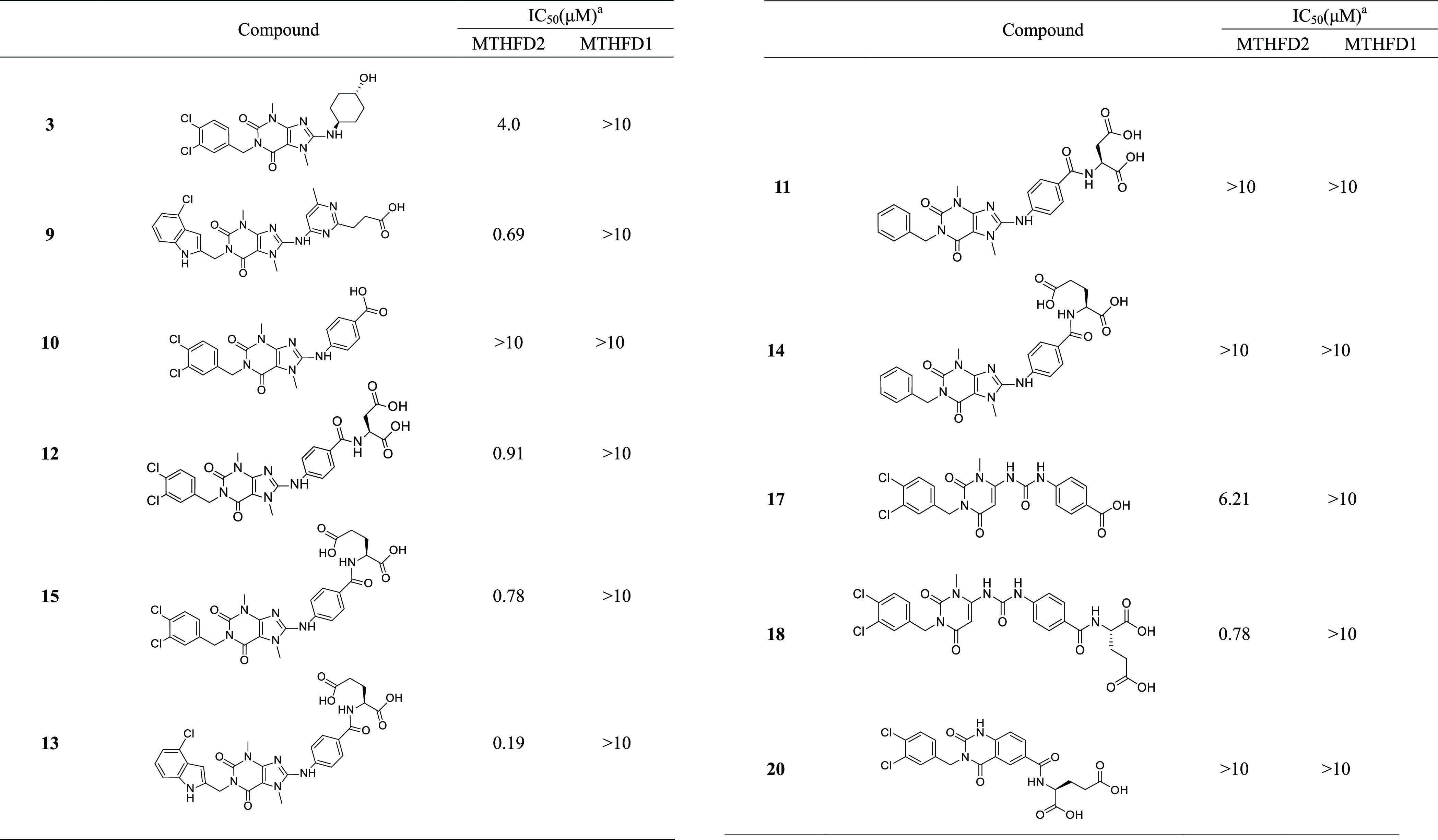
Inhibition of MTHFD2 by Xanthine Derivatives

a Values of IC_50_ are expressed as the mean of three independent experiments and are within ±15%.

Replacement of the *trans*-4-aminocyclohexanol group of **3** with 4-aminobenzoic acid to give **10** decreased the inhibitory activity against MTHFD2 (IC_50_ > 10 μM). Addition of aspartic acid and glutamic acid by their condensation with the carboxyl group of 4-aminobenzoic acid (**10**) gave compounds **12** and **15**, respectively, which showed significantly improved potency compared to **10** (IC_50_ values of 0.91 and 0.78 μM, respectively). Next, modifications to the 3,4-dichlorobenzyl moiety at the *N*1-position of the xanthine core were examined. Replacement of the 3,4-dichlorobenzyl moiety of **15** with a 4-chloro-2-chloromethyl-1*H*-indole group (**13**) led to a further fourfold improvement in potency. In contrast, when the 3,4-dichlorobenzyl moiety of **15** was replaced with an unsubstituted benzyl group to generate **14**, the inhibitory activity was completely lost. Similarly, the potency of unsubstituted benzyl **11** was also dramatically decreased compared to that of its dichlorobenzyl analogue **12**.

In addition to evaluating the effects of the terminal amino acids and substituents at the *N*1-position of xanthine, the xanthine core was also modified. When the fused heterobicyclic xanthine (**10** or **15**) was replaced with heterocyclic pyrimidinedione (**17** or **18**), pyrimidinedione **17** with a urea moiety showed a weak MTHFD2 inhibitory effect (IC_50_ = 6.21 μM) but was a more potent inhibitor than xanthine **10** (IC_50_ > 10 μM). Pyrimidinedione **18** exhibited submicromolar inhibitory activity against MTHFD2 (IC_50_ = 0.78 μM), similar to that of xanthine **15**. A significant drop in potency was observed when the xanthine of **15** was replaced with the quinazoline-2,4-dione moiety, and the aniline group of **15** was removed to give **20**.

It is worth noting that all of the inhibitors of MTHFD2 depicted in Table 1 were highly selective; none of them exhibited activity against MTHFD1 (IC_50_ > 10 μM).

### Structural Biology Study of **15** Bound to MTHFD2

**Figure 2:**
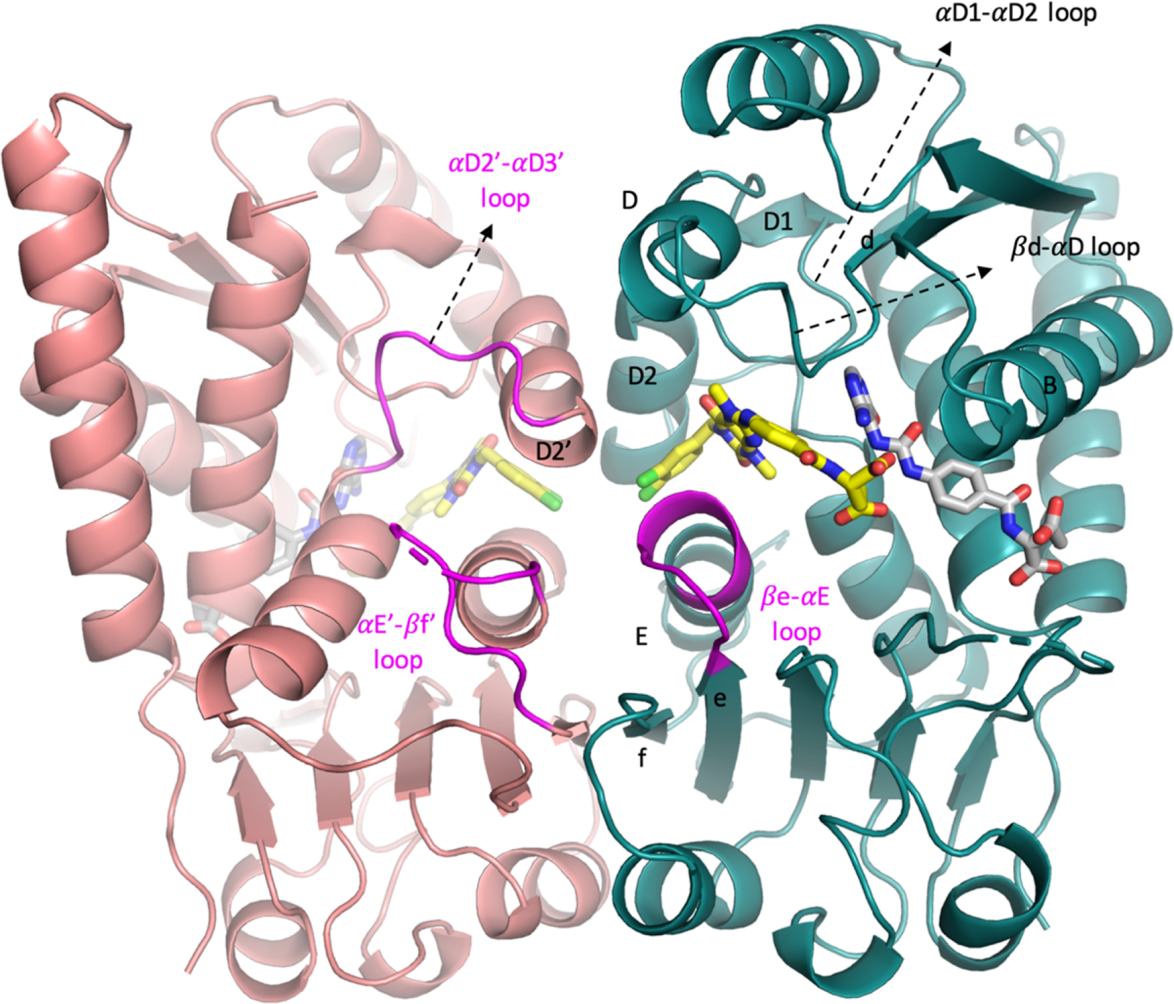
Overall structure of MTHFD2/**15**/**21** (PDB 7EHM). Monomer A is depicted in teal and monomer B in pink. Helices B, D, D1, D2, D2′, and E and the β-sheets e and f are marked. **15** (xanthine derivative) and **21** (folate analogue) are shown in yellow and gray, respectively. The βe-αE loop, αD2′-αD3′ loop, and αE′-βf′ loop are also highlighted in magenta.

To further elucidate the binding mode and obtain detailed structural insights into the interactions between xanthine derivatives and MTHFD2, the structural biology study of **15** bound to MTHFD2 was performed. The ternary structure of the human MTHFD2 bound to compound **15** and **21** (a folate analogue, Figure S-1) was determined at a resolution of 2.13 Å (Figure 2) (the ternary structure is hereafter referred to as MTHFD2/**15**/**21**). Data collection and refinement statistics are summarized in Table 2. The complex structure contains one dimer (monomer A and monomer B) of MTHFD2 in the asymmetric unit, and the density map of MTHFD2 is clear except for residues Ala218-Arg221 and His280-Lys286 which are flexible and missing in the protein structure. **21** is a folate analogue with weak inhibition (IC_50_ = 8.33 μM) and binds to the substrate-binding site. Compound **15** occupies a pocket near the substrate-binding site and coexists with **21**. **15** is located between the helix D2 and E in the dimer interface and surrounded by helix D, helix B, the βd-αD loop, and αD1-αD2 loop. Several conformational changes upon the binding of **15** were observed, including the shift of the βe-αE loop (amino acids 199–206 in monomer A) toward the C-lobe, destabilization of the αE′-βf′ loop (amino acids 214–227 in monomer B), and the disruption of the dimer interactions (Figure 2). These conformational changes subsequently obstruct the binding of cofactor NAD^+^ and phosphate to MTHFD2.

**Table 2:** X-ray Diffraction Data and Structure Refinement Statistics for MTHFD2 Complex Structures

	MTHFD2/NAD^+^/P_i_/**21** (7EHJ)	MTHFD2/**15**/**21** (7EHM)	MTHFD2/**9**/**21** (7EHN)	MTHFD2/**3**/**21** (7EHV)
resolution (Å)	26.85–2.16	24.92–2.13	25.39–2.25	27.34–2.61
space group	*P*6_5_	*P*6_5_	*P*6_5_	*P*6_5_
unit cell				
*a* = *b*, *c* (Å)	115.06, 113.56	114.23, 114.16	113.17, 113.67	112.89, 113.88
(α = β = 90°, γ = 120°)				
unique reflections	45929 (4172)	47264 (4707)	39045 (3829)	25010 (2502)
*I*/σ	15.56 (2.17)	21.34 (2.23)	14.12 (2.12)	14.06 (4.03)
*R*_merge_ (%)	8.2 (41.1)	6.1 (55.5)	7.6 (52.2)	7.7 (26.5)
completeness (%)	98.8 (91.6)	99.9 (100.0)	98.7 (97.7)	99.6 (100.0)
*R*_work_/*R*_free_	0.1915/0.2313	0.1954/0.2269	0.2005/0.2398	0.1930/0.2357
r.m.s.d (bond) (Å)	0.010	0.009	0.009	0.010
r.m.s.d (angle) (deg)	1.160	1.067	1.158	1.169
Ramachandran favored (%)	98.95	99.28	98.92	98.02

**Figure 3:**
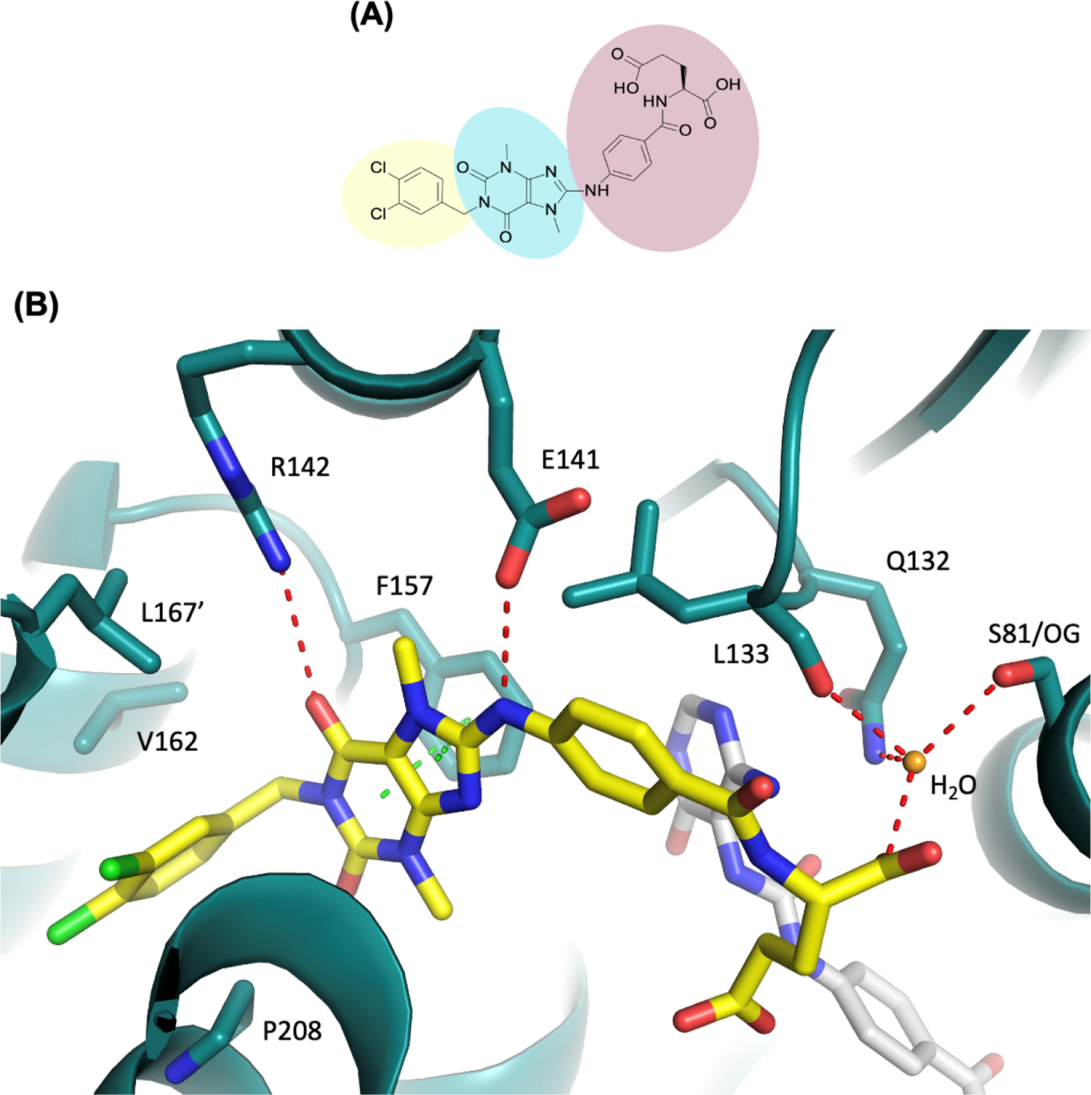
(A) Chemical structure of **15**. **15** contains a hydrophobic head group (yellow), core region (blue), and the tail group (pink). (B) Crystal structure of MTHFD2 (deep teal) in complex with **15** (yellow) and **21** (gray) (PDB 7EHM). Hydrogen-bond and π–π interactions are shown in red and green dashed lines, respectively. The water molecule is shown as a sphere (bright orange). The tail of **15** forms a hydrogen-bond network with the side chains of Ser81 (S81/OG), Gln132, and the main chain of Leu133 bridged with a water molecule.

**15** (Figure 3A) consists of a hydrophobic head (1,2-dichlorobenzene), a core region (1,3,7-trimethyl-3,7-dihydro-1*H*-purine-2,6-dione), and a tail ((4-amino-benzoyl)-l-glutamic acid). The hydrophobic head extends into the dimer interface and is positioned in the hydrophobic pocket formed by helix D2 and E in monomer A and helix D2′ in monomer B (Figure 2). This pocket consists of various hydrophobic amino acids, including Val162, Met165, Pro174, Met207, Pro208, and Met211 in monomer A and Leu167′ in monomer B. The hydrophobic head of **15** forms a shape complementarity contact in this pocket and makes hydrophobic interactions with Val162, Pro208 in monomer A, and Leu167′ in monomer B. The 6-carbonyl group of the core region is hydrogen bonded with the side chain of Arg142 and the amino group adjacent to the core region forms a hydrogen bond with the side chain of Glu141. The purine moiety of the core also forms extensive π–π interactions with Phe157 (Figure 3B). The tail part consists of an amino-benzoyl moiety connected with l-glutamic acid. The benzoyl moiety of the tail makes hydrophobic interactions with Leu133. The α-carboxyl group of glutamic acid forms an extensive hydrogen-bond network with the side chains of Ser81, Gln132, and the main chain of Leu133 bridged by a water molecule (Figure 3B). The γ-carboxyl group of glutamic acid is more flexible than the α-carboxyl group and might form a hydrogen bond with **21** in the substrate-binding site. Our previous SAR analysis (Table 1) revealed that substitution of the l-glutamic acid moiety of **15** with the shorter l-aspartic acid moiety (**12**) maintained the inhibitory activity, indicating the replacement of the tail with a moiety capable of hydrogen bond formation to be practical.

### Structure-Based Drug Design of Xanthine Derivatives

**Figure 4:**
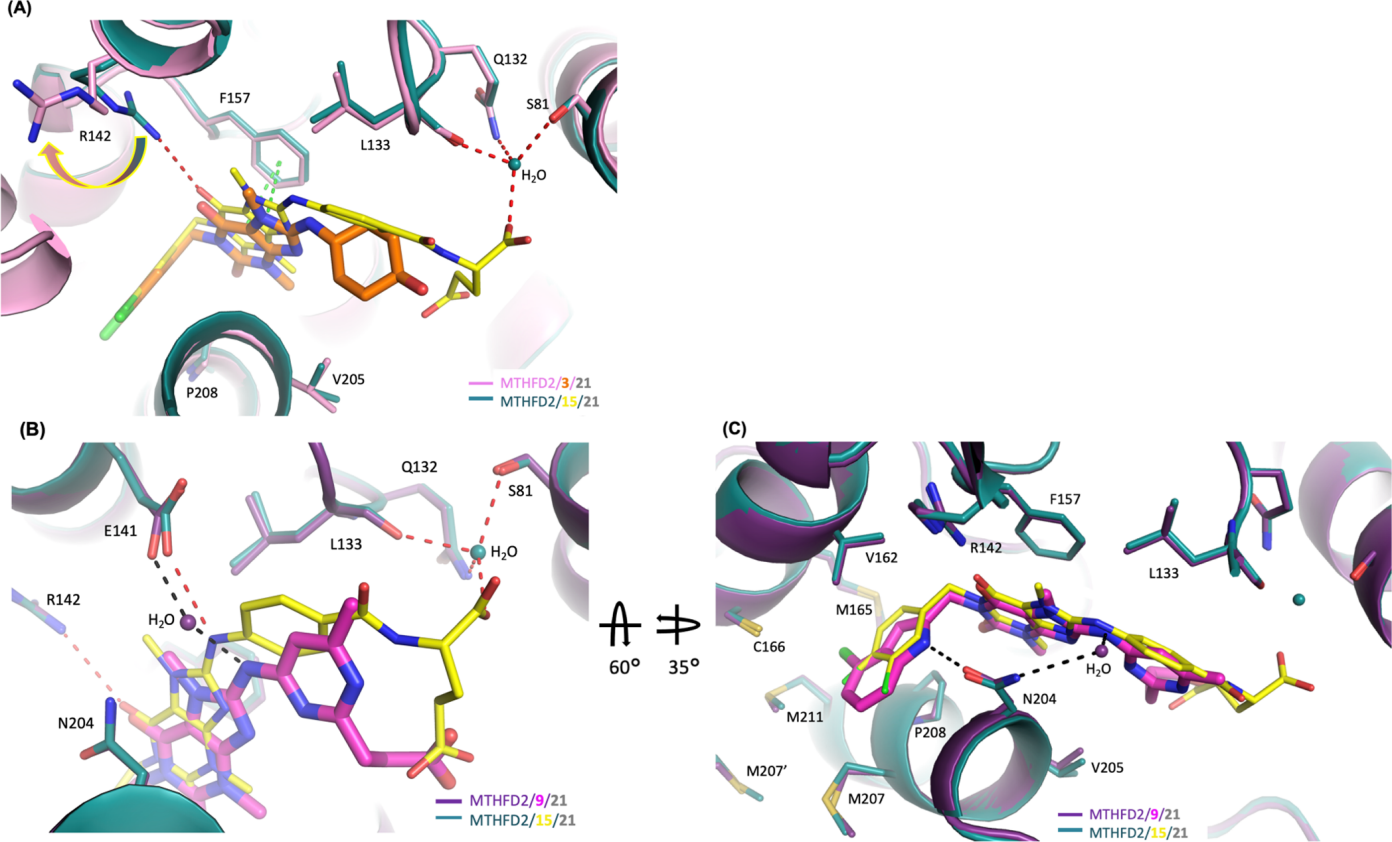
(A) Superimposition of the crystal structure of MTHFD2/**15**/**21** (PDB 7EHM) on MTHFD2/**3**/**21** (PDB 7EHV). For clarity, compound **21** is omitted in the figure. Red dashed lines indicate the hydrogen-bond and green ones indicate the π–π interactions. The tail group of **3** loses the important hydrogen-bond network with MTHFD2 in comparison with **15** and therefore weakens its inhibition toward MTHFD2. (B) and (C) Superimposition of the crystal structure of MTHFD2/**15**/**21** (PDB 7EHM) on MTHFD2/**9**/**21** (PDB 7EHN). (C) is the view of (B) after 60° rotation along the *X*-axis followed by 35° rotation along the *Y*-axis. For clarity, compound **21** is omitted in the figure. Red and black dashed lines indicate the hydrogen bond. In (B), the tail group of **9** loses the hydrogen-bond network with MTHFD2 and, instead of direct interactions with Glu141 as observed in **15**, the core of **9** forms the hydrogen-bond interaction with Glu141 through a water molecule. In (C), in comparison with **15** (yellow), the indole group of **9** (magenta) forms shape complementarity interactions with MTHFD2 and makes the additional hydrogen-bond interaction with Asn204.

To further investigate SAR at the molecular level, structural biology studies of **3** and **9** were also performed and all structures compared. The structure of MTHFD2 in complex with **3** was solved to 2.61 Å and it also coexisted with **21** (the ternary structure is hereafter referred to as MTHFD2/**3**/**21**). Comparing the structure of MTHFD2/**3**/**21** with that of MTHFD2/**15**/**21** revealed that the hydrophobic head of the two structures superimposed well (Figure 4A). The most significant difference between the two was the lack of the glutamic acid tail group in **3**, precluding its formation of extensive hydrogen-bond networks with the protein and greatly diminishing activity against MTHFD2 (Figure 4A). Furthermore, without the interactions of the tail group with MTHFD2, compound **3** was not held in the proper position to form the optimum interactions with the protein. Particularly, the cyclohexanol moiety of **3** shifted away with loss of hydrophobic interactions with Leu133. In addition, the core group of **3** slightly moved away from helix D, weakening interactions with Phe157 and Arg142. Arg142 could no longer form the hydrogen bond with **3** and the flexible side chain of Arg142 shifted away (Figure 4A). All of these structural differences as compared with MTHFD2/**3**/**21** and MTHFD2/**15**/**21** explain the decreased inhibitory activity of **3**.

The structural biology and SAR studies (Table 1) of the xanthine derivatives both revealed the importance of the tail group for MTHFD2 inhibitory activity. For example, **10**, which lacked the l-glutamic group, exhibited significantly decreased inhibitory activity compared to **15**, suggesting that the hydrogen-bond interaction network established between the tail group and MTHFD2 greatly contributed to the activity of **15**. Moreover, the removal of the aniline group in **20** as compared with **15** resulted in the loss of activity. The reason for the decreased activity of **20** could be that the shorter substituent length restricted the orientation of the l-glutamic group to form the optimal interactions with the surrounding residues. Furthermore, the loss of the hydrophobic interactions of **20** with Leu133 due to the removal of the aniline group might also account for the decreased activity of **20**.

Compound **9** and **15** show similar activities, despite having different chemical structures and, in particular, different tail moieties: whereas **15** bears a bulky glutamic moiety, **9** carries a relatively small propanoic moiety. To compare the interactions of these two compounds with MTHFD2, the structure of MTHFD2 in complex with **9** was solved to 2.25 Å (the ternary structure is hereafter referred to as MTHFD2/**9**/**21**) and compared with the MTHFD2/**15**/**21** structure previously obtained. The propanoic acid group of **9** occupied a similar position to the γ-carboxyl group of glutamic acid in **15**. However, the α-carboxyl group of **15** is absent in **9** and thus loses the hydrogen interaction network with Ser81, Gln132, and Leu133 (Figure 4B). Also, the core of **9** was observed to be shifted away from helix D, resulting in the loss of a hydrogen bond with Arg142. In addition, instead of direct interactions with Glu141 of MTHFD2 as observed with **15**, **9** was found to interact with Glu141 through a water molecule (Figure 4B).

**9** and **15** also have different head moieties: whereas, **15** bears a phenyl-based head, **9** has an indole moiety consisting of two fused rings. The indole moiety was found to extend further into the dimer interface compared to the phenyl-based head, leading to the formation of stronger shape complementarity interactions with MTHFD2 and the additional hydrophobic interactions with Lys203 and Met207 (Figure 4C). Most importantly, the nitrogen atom on the indole moiety of **9** was hydrogen bonded with the side chain of Asn204, which is absent in the structure of **15** bound to MTHFD2. The more extensive shape complementarity interactions and the additional hydrogen-bond interaction around the head group of **9** compensate for the losses of hydrogen-bond networks observed in the tail group of **15**, resulting in the comparable activity of these two inhibitors.

### Uncompetitive Inhibition of Xanthine Derivatives

**Figure 5:**
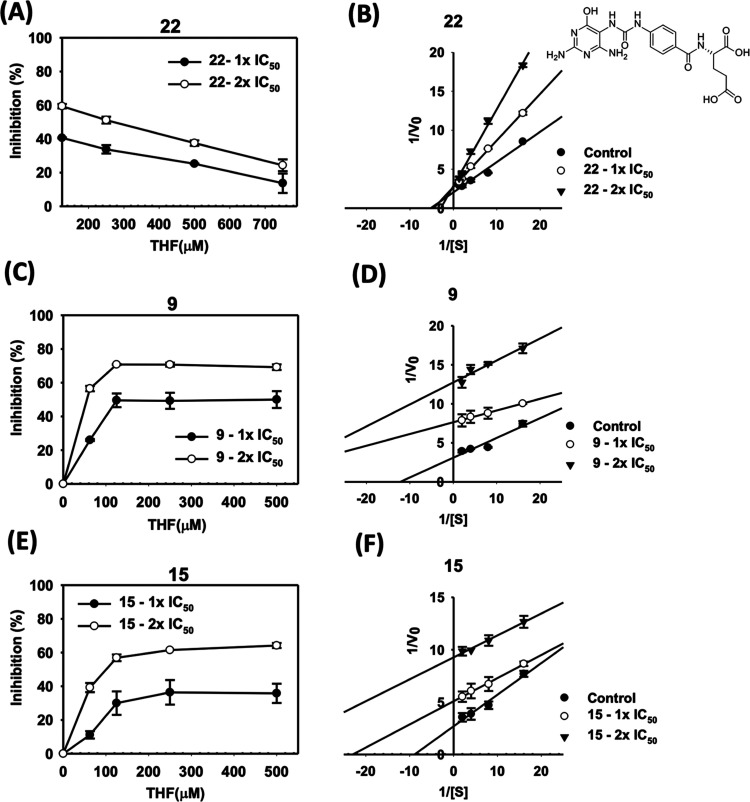
Analysis of the inhibition kinetics of human MTHFD2 by **22**, **9**, and **15**. The test concentrations of MTHFD2 inhibitors were 1× IC_50_ and 2× IC_50_ values obtained from the enzyme inhibition assay. Effects of **22** (A), **9** (C), and **15** (E) on the enzyme activity of MTHFD2 under different substrate concentrations. The double-reciprocal plot analysis was used to demonstrate the inhibition kinetics of human MTHFD2 by **22** (B), **9** (D), and **15** (F).

To elucidate the mechanism of the enzymatic inhibition, kinetic studies of inhibition of human MTHFD2 by 2-(4-(3-(2,4-diamino-6-oxo-1,6-dihydropyrimidin-5-yl)ureido)benzamido)pentanedioic acid^27^ (**22**), **9**, and **15** were performed (Figure 5). The recombinant human MTHFD2 was prepared by the insect cell expression system. Enzymatic activity of MTHFD2 was examined after treatment of **22**, **9**, and **15**. The chemical structure of **22** resembles tetrahydrofolate (THF) and occupies the substrate-binding site.^27^ It was found that the degree of catalytic inhibition of MTHFD2 could be relieved by increasing the substrate concentration at any fixed concentration of **22**, a substrate-based inhibitor (Figure 5A). In addition, the *K*_m_ was increased and *V*_max_ remained the same based on the double-reciprocal plot (Figure 5B), indicating that **22** inhibited MTHFD2 in a competitive manner.

In contrast, the mechanism of inhibition of MTHFD2 by xanthine derivatives, including **9** and **15**, is quite different from that of a folatelike inhibitor. At any given concentration of **9** and **15**, the degree of inhibition of MTHFD2 activity increased with increasing substrate concentration until no further increase was observed when the substrate fully occupied the enzyme binding site (Figure 5C,E).

In addition, a double-reciprocal plot showed a pattern of parallel lines at all inhibitor concentrations tested wherein both *K*_m_ and *V*_max_ decreased as the concentrations of **9** and **15** increased, indicating that the inhibitory mechanism of MTHFD2 by **9** and **15** was uncompetitive (Figure 5D,F). The main characteristic of the uncompetitive inhibition is that the inhibitor binds only to the enzyme–substrate complex but not to the free enzyme, thus forming a catalytically inactive ternary enzyme–substrate–inhibitor complex which greatly slows product formation.^28^ Taken together, these results suggest that **22** competes with the substrate and binds to MTHFD2 at the substrate site. However, xanthine derivatives, including **9** and **15**, coexist with the substrate and bind to a nonsubstrate-binding site of MTHFD2.

### Conformational Changes Induced by **15**

**Figure 6:**
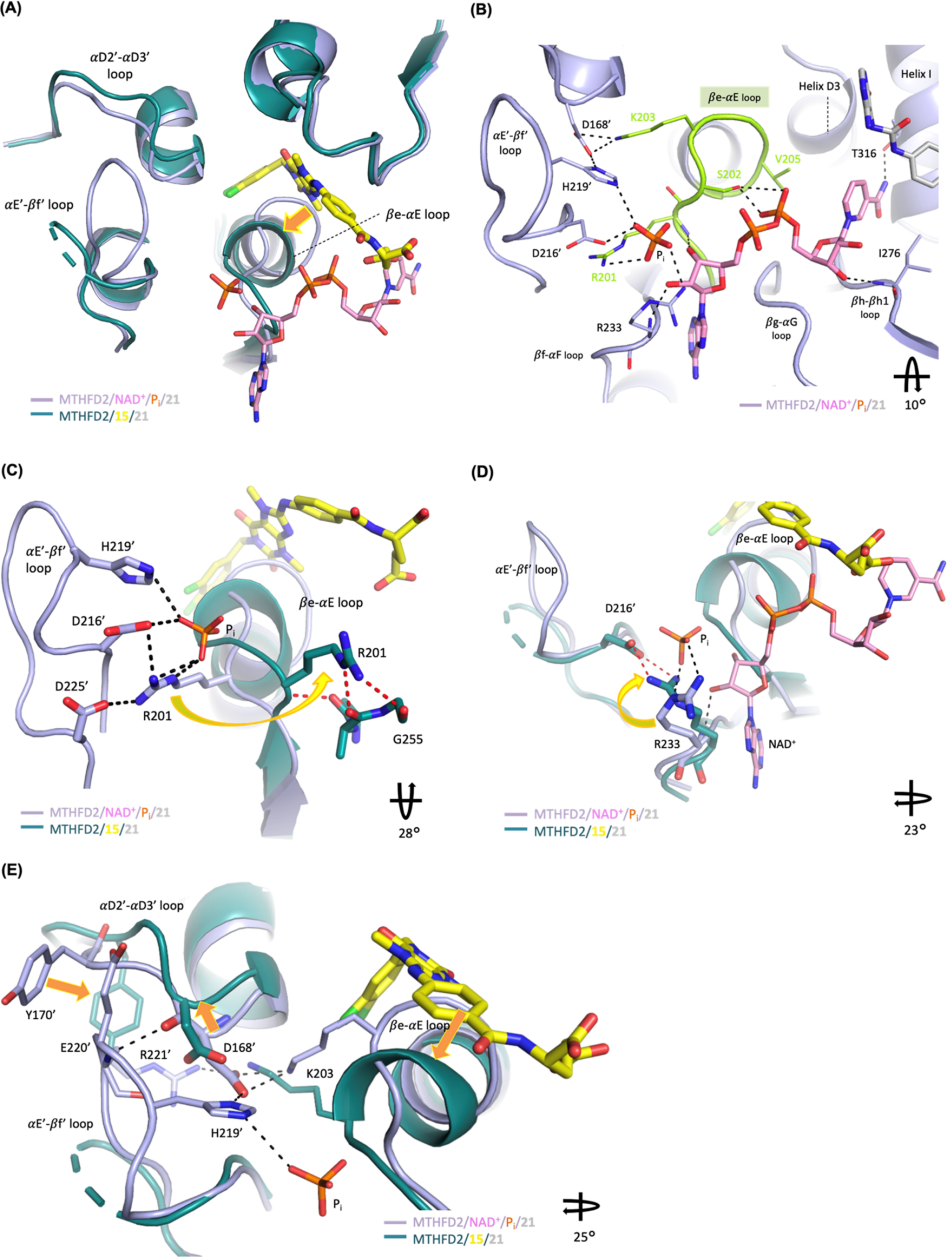
Superimposition of the crystal structure of MTHFD2/**15**/**21** (PDB 7EHM) on MTHFD2/NAD^+^/P_i_/**21** (PDB 7EHJ). For clarity, compound **21** is omitted in the figure. (A) Upon the binding of **15**, MTHFD2 undergoes dramatic conformational changes, including the shift of the βe-αE loop in monomer A, destabilization of the αE′-βf′ loop, and movement of the αD2′-αD3′ loop in monomer B. The orange arrow highlights the shift of the βe-αE loop upon the binding of **15**. (B) NAD^+^ and the phosphate ion form interactions with the Rossmann fold motif in the βe-αE loop (lime green) and surrounding residues. (C) and (D) In the presence of **15**, Arg201 and Arg233 shift away from their coordinating position and are unable to bind to NAD^+^ and the phosphate ion. (E) Conformational change induced by **15** consequently shifts the αD2′-αD3′ loop in monomer B away from its original position and therefore disrupts its interactions with the αE′-βf′ loop, leading to the destabilization of the αE′-βf′ loop. The orange arrows highlight the shifts of the βe-αE loop, αD2′-αD3′ loop, and Tyr170′ upon the binding of **15**. The specified rotations along the drawn axis, relative to Figure 6A, are shown in the right bottom of Figure 6B–E.

Superimposition of the MTHFD2/**15**/**21** complex structure on the MTHFD2/NAD^+^/P_i_/**21** complex structure reveals that upon the binding of **15**, MTHFD2 induces dramatic conformational changes, including the shift of the βe-αE loop in monomer A, destabilization of the αE′-βf′ loop (Figure 6A), and movement of the αD2′-αD3′ loop in monomer B. The structure of MTHFD2/NAD^+^/P_i_/**21** was obtained by cocrystallization of MTHFD2 with NAD^+^, phosphate (P_i_), and **21**. **21** binds to the substrate-binding site while NAD^+^ is located between the N and C-lobes and surrounded by the βe-αE, βf-αF, βg-αG, and βh-βh1 loops, helix D3, and helix I (Figure 6B). Of these secondary structures, the βe-αE loop consists of a G^200^RSKNVG motif, a conserved fingerprint pattern observed in the Rossmann fold of the MTHFD family.^11^ The Rossmann fold is a well-known supersecondary structure of dinucleotide-binding enzymes and is a characteristic fold of the cofactor (NAD(P)^+^) binding.^29^ As revealed in the MTHFD2/NAD^+^/P_i_/**21** structure, NAD^+^ is hydrogen bonded to Arg201 and Ser202 on the βe-αE loop (Figure 6B). In addition, NAD^+^ also forms hydrophobic interactions with Thr176 and Val205 and hydrogen-bond interactions with Arg233, Ile276, and Thr316 (Figure 6B). The phosphate is located in the dimer interface, forming an extensive hydrogen-bond network with Arg201 and Arg233 in monomer A and Asp216′ and His219′ in monomer B.

Binding of **15** to MTHFD2 causes the βe-αE loop to shift toward the C-lobe to accommodate **15** and, surprisingly, the important conserved motif of the Rossmann fold undergoes a conformational change from a loop to an extended helix, the extension of helix E. The conformational change of the βe-αE loop consequently eliminates the interaction network between NAD^+^, phosphate, and MTHFD2 (Figure 6A,B).

Furthermore, Arg201, located in the βe-αE loop, plays an important role in phosphate binding and contributes to the activity of human MTHFD2 as the mutation of Arg201 to lysine resulted in the total loss of enzymatic activity.^14^ In the presence of NAD^+^, Arg201 serves as the interaction center to form hydrogen bonds with the phosphate ion, the adenosine ribose ring of NAD^+^, and also coordinates with Asp216′, His219′, and Asp225′ in monomer B through the salt bridge and hydrogen-bond network (Figure 6B,C). However, in the presence of **15**, Arg201 shifts away from its coordinating position and is unable to bind to the cofactor and phosphate (Figure 6C). It exposes to the solvent accessible area and becomes more flexible with less interactions with surrounding residues.

Arg233, close to the βe-αE loop, also undergoes conformational changes upon inhibitor binding (Figure 6D). In the MTHFD2/NAD^+^/P_i_/**21** structure, Arg233 binds to the phosphate ion and NAD^+^, providing evidence that Arg233 contributes to the phosphate binding and assists in positioning NAD^+^. Studies also showed that the mutation of Arg233 resulted in the decreased activity of MTHFD2,^30^ supporting the biological role of Arg233 in regulating MTHFD2 activity. Superimposition of the structure of MTHFD2/**15**/**21** on that of MTHFD2/NAD^+^/P_i_/**21** reveals that Arg233 no longer interacts with the cofactor and phosphate and is orientated differently to form the salt bridge with the side chain of Asp216′ in monomer B (Figure 6D).

In conclusion, the conformational change of the βe-αE loop and the movement of Arg201 and Arg233 upon the binding of **15** account for its allosteric inhibition of MTHFD2.

Furthermore, the αD2′-αD3′ and αE′-βf′ loops are also perturbed by inhibitor binding. The conformational change induced by **15** consequently shifts the αD2′-αD3′ loop in monomer B away from its original position and therefore destabilizes the αE′-βf′ loop in monomer B (Figure 6E). The αE′-βf′ loop becomes very flexible and residues of 218-221 are invisible in the crystal structure.

In the absence of **15**, the αD2′-αD3′ loop stabilizes the αE′-βf′ loop via an extensive interaction network. For examples, Asp168′ in the αD2′-αD3′ loop forms the strong hydrogen-bond interactions with Arg221′ and His219′ in the αE′-βf′ loop. However, in the presence of **15**, Asp168′ moves upward and the extensive interaction network with Arg221′ and His219′ is lost (Figure 6E). In addition, Tyr170′ in the αD2′-αD3′ loop forms interactions with Glu220′ in the αE′-βf′ loop. However, the binding of **15** moves Tyr170′, disrupting its interactions with Glu220′ and consequently weakening the interactions between the αD2′-αD3′ loop and the αE′-βf′ loop. Furthermore, in the absence of **15**, His219′ in the αE′-βf′ loop binds to phosphate. However, in the presence of **15**, the phosphate ion can no longer bind to MTHFD2 and therefore is unable to stabilize the αE′-βf′ loop, particularly through the interaction with His219′.

Previous reports showed that Arg168 and His219 contribute to Mg^2+^ binding and Asp225 from another monomer helps to hold Arg201 in place.^30^ In addition to contributing to the dimer interactions, these residues also play roles in maintaining the activity of MTHFD2.

## Discussion and Conclusions

In this study, the mechanistic basis for the allosteric inhibition of MTHFD2 by xanthine derivative **15** has been elucidated based on the inhibitor-bound MTHFD2 structure, which was solved at a resolution of 2.13 Å. The density map clearly shows **15** to occupy a novel allosteric site close to the canonical substrate-binding pocket. This binding mechanism is a unique characteristic of **15**, as all other known inhibitors targeting the MTHFD family are competitive and bind to the substrate site. Our structural biology study also reveals that **15** coexists with **21**, a folate analogue binding to the substrate site. This is consistent with the results of our kinetic study, which found that the inhibition of MTHFD2 by **15** was uncompetitive.

Several studies have identified the benefits of allosteric inhibitors for the purpose of drug design. First, the allosteric binding site is usually less conserved than the substrate-binding site among the homologous family enzymes, and therefore allosteric inhibitors are often more selective than nonallosteric inhibitors and associated with fewer off-target side effects.^31^ For example, Bagal et al. reported a series of allosteric tropomyosin receptor kinase subtype A (TRKA) inhibitors and these inhibitors showed high selectivity over subtypes, TRKB and TRKC. Structural biology studies revealed that the interactions of the inhibitors with the poorly conserved juxtamembrane (JM) region contributed to their selectivity.^32^ Friberg et al. reported a series of lactate dehydrogenase A (LDHA) inhibitors with selectivity over their isoenzyme, LDHB. These inhibitors bound to an allosteric site located at the interface between two monomers, a region less conserved compared with the cofactor and pyruvate-binding sites.^33^ In our study, the xanthine derivatives were highly selective for MTHFD2 over MTHFD1. They bound to an allosteric site with a low sequence identity between MTHFD2 and MTHFD1. In particular, several key residues of MTHFD2 interacting with compound **15**, including Phe157 (Leu127 in MTHFD1), Glu141 (Thr111 in MTHFD1), and Arg142 (Glu112 in MTHFD1) are not conserved and would attribute to the selectivity. For example, replacement of Phe157 in MTHFD2 with Leu127 in MTHFD1 would result in the loss of important π–π interactions with the purine moiety of the xanthine core. In addition, the αE′-βf′ loop, the βe-αE loop, and the αD2′-αD3′ loop, which undergo conformational changes upon the binding of **15**, are poorly conserved in the MTHFD family. All of these results help explain the selectivity of the xanthine derivatives for MTHFD2 over MTHFD1.

Second, allosteric inhibition could offer a less disruptive way to influence the functioning of a pathway and may overcome the problem of drug resistance, which usually arises from mutations at the substrate-binding site.

Even more interestingly, structural biology results demonstrated that **15** induced dramatic conformational changes in MTHFD2 and consequently disrupted interactions among MTHFD2, the cofactor (NAD^+^), and phosphate. When **15** bound to MTHFD2, the βe-αE loop shifted toward the C-lobe to accommodate **15** and, surprisingly, the conserved motif of the Rossmann fold, a key motif in the NAD^+^ binding loop, underwent a conformational change from a loop to an extended helix. Subsequently, Arg201 and Arg233 shifted away from their coordinating positions and were unable to bind to the cofactor and phosphate.

Finally, as revealed by the structure of **15** bound to MTHFD2, the α-carboxyl group of the glutamic acid moiety formed the extensive hydrogen-bond network with MTHFD2, whereas the γ-carboxyl group of glutamic acid was more flexible without strong interactions with the surrounding residues. Therefore, it is suggested that the glutamic acid moiety of **15** would be replaced with the amino acids containing the aliphatic side chain, such as leucine, isoleucine, or valine, to optimize the lipophilicity and reduce the molecular weight to improve physicochemical properties. Moreover, replacement of the glutamic acid moiety with heteroaryl amines, such as 1*H*-tetrazol-5-ylamine, [1,3,4]thiadiazol-2-ylamine and 1*H*-pyrazol-3-ylamine, would expand the chemical diversity as well as maintain the important hydrogen-bond network.

In summary, to the best of our knowledge, this is the first study to identify the allosteric inhibitors targeting the MTHFD family and directly observe allosteric binding at the molecular level by the structural biology study. Our results are expected to be useful for inhibition mechanism studies of the MTHFD protein family and should provide insights into the development of the novel MTHFD inhibitors for cancer treatment.

## Experimental Section

### Chemical Methods

All commercial chemicals and solvents are of reagent grade and used without further treatment, unless otherwise noted. ^1^H NMR spectra were acquired on a Varian Mercury-300 or Mercury-400 spectrometer. Chemical shifts are reported in parts per million (ppm, δ), relative to the solvent peak or TMS. LC/MS data were measured on an Agilent MSD-1100 ESI–MS/MS system. High-resolution mass spectra (HRMS) were measured with a Thermo Finnigan (TSQ Quantum) electrospray ionization (ESI) mass spectrometer. Flash column chromatography was done using silica gel (Merck Kieselgel 60, No. 9385, 230–400 mesh ASTM). Reactions were monitored by TLC using Merck 60 F_254_ silica gel glass-backed plates (5 × 10 cm); zones were detected visually under ultraviolet irradiation (254 nm) or by spraying with the phosphomolybdic acid reagent (Aldrich) followed by heating at 80 °C. All starting materials and amines were commercially available unless otherwise indicated. The purity of compounds was determined by a Hitachi 2000 series HPLC system based on a reverse phase C_18_ column (Agilent ZORBAX Eclipse XDB-C18 5 μm, 4.6 mm × 150 mm) under the following gradient elution conditions: Mobile phase A-acetonitrile (10% to 90%, 0 to 45 min) and mobile phase B-2 mM NH_4_OAc aqueous solution containing 0.1% formic acid (90% to 10%, 0 to 60 min). The flow-rate was 0.5 mL/min and the injection volume was 20 μL. The system operated at 25 °C. Peaks were detected at λ = 254 nm. Purity of all of the tested compounds were found to be >95% except for compound 20 (91.9%).

#### 1-(3,4-Dichlorobenzyl)-8-[(*trans*-4-hydroxycyclohexyl)amino]-3,7-dimethyl-3,7-dihydro-1*H*-purine-2,6-dione (**3**)

^1^H NMR (400 MHz, DMSO-*d*_6_): δ 7.53 (d, *J* = 8.0 Hz, 1H), 7.49 (s, 1H), 7.23 (d, *J* = 8.4 Hz, 1H), 6.75 (d, *J* = 7.6 Hz, 1H), 4.95 (s, 2H), 4.55 (d, *J* = 4.4 Hz, 1H), 3.63–3.45 (m, 1H), 3.52 (s, 3H), 3.42–3.30 (m, 1H), 3.32 (s, 3H), 1.98–1.88 (m, 2H), 1.88–1.78 (m, 2H), 1.41–1.30 (m, 2H), 1.30–1.18 (m, 2H); ^13^C NMR (75 MHz, DMSO-*d*_6_): δ 154.2, 152.8, 151.3, 149.4, 139.9, 131.2, 130.8, 130.0, 129.9, 128.4, 102.1, 68.8, 51.6, 42.7, 34.6, 31.1, 30.3, 29.8; MS (ES^+^) *m*/*z* calcd for C_20_H_23_Cl_2_N_5_O_3_: 451.1; found: 452.4 [M + H]^+;^ HRMS (ESI^+^) calcd for C_20_H_24_Cl_2_N_5_O_3_: 452.1256; found: 452.1257 [M + H]^+^; HPLC *t*_R_ = 20.12 min, 96.6%.

#### 3-[4-({1-[(4-Chloro-1*H*-indol-2-yl)methyl]-3,7-dimethyl-2,6-dioxo-2,3,6,7-tetrahydro-1*H*-purin-8-yl}amino)-6-methylpyrimidin-2-yl]propanoic Acid (**9**)

^1^H NMR (400 MHz, DMSO-*d*_6_): δ 11.26 (s, 1H), 7.32–7.29 (m, 2H), 7.01–6.97 (m, 2H), 6.26 (s, 1H), 5.19 (s, 2H), 3.76 (s, 3H), 3.45 (s, 3H), 2.90 (d, *J* = 7.0 Hz, 2H), 2.66 (d, *J* = 7.0 Hz, 2H), 2.32 (s, 3H); ^13^C NMR (75 MHz, DMSO-*d*_6_): δ 174.5, 167.9, 166.7, 159.5, 153.9, 151.2, 147.9, 147.5, 137.3, 137.0, 126.7, 124.0, 122.0, 118.9, 110.9, 104.1, 98.1, 38.0, 33.5, 32.0, 31.7, 30.1, 24.4; MS (ES^+^) *m*/*z* calcd for C_24_H_23_ClN_8_O_4_: 522.2; found: 523.2 [M + H]^+;^ HRMS (ESI^+^) calcd for C_24_H_24_ClN_8_O_4_: 523.1609; found: 523.1611 [M + H]^+^; HPLC *t*_R_ = 18.35 min, 99.9%.

#### 4-{[1-(3,4-Dichlorobenzyl)-3,7-dimethyl-2,6-dioxo-2,3,6,7-tetrahydro-1*H*-purin-8-yl]amino}benzoic Acid (**10**)

^1^H NMR (400 MHz, DMSO-*d*_6_): δ 12.58 (bs, 1H), 9.55 (s, 1H), 7.88 (d, *J* = 8.8 Hz, 2H), 7.76 (d, *J* = 8.8 Hz, 2H), 7.55 (d, *J* = 8.4 Hz, 1H), 7.54 (s, 1H), 7.27 (dd, *J* = 8.0, 2.0 Hz, 1H), 5.01 (s, 2H), 3.79 (s, 3H), 3.42 (s, 3H); ^13^C NMR (75 MHz, DMSO-*d*_6_): δ 167.5, 153.7, 151.3, 149.1, 148.0, 144.6, 139.5, 131.2, 130.9, 130.1, 128.4, 123.9, 117.5, 102.9, 42.9, 31.3, 30.0; MS (ES^–^) *m*/*z* calcd for C_21_H_17_Cl_2_N_5_O_4_: 473.1; found: 472.0 [M – H]^−^; HRMS (ESI^–^) calcd for C_21_H_16_Cl_2_N_5_O_4_: 472.0579; found: 472.0579 [M – H]^−;^ HPLC *t*_R_ = 23.01 min, 95.5%.

#### *N*-{4-[(1-Benzyl-3,7-dimethyl-2,6-dioxo-2,3,6,7-tetrahydro-1*H*-purin-8-yl)amino]benzoyl}-l-aspartic Acid (**11**)

^1^H NMR (400 MHz, DMSO-*d*_6_): δ 12.68 (bs, 2H), 9.48 (s, 1H), 8.54 (d, *J* = 7.6 Hz, 1H), 7.82 (d, *J* = 8.8 Hz, 2H), 7.75 (d, *J* = 8.4 Hz, 2H), 7.29–7.22 (m, 4H), 7.21 (t, *J* = 8.8 Hz, 1H), 5.03 (s, 2H), 4.73–4.69 (m, 1H), 3.80 (s, 3H), 3.42 (s, 3H), 2.81 (dd, *J* = 16.0, 6.0 Hz, 1H), 2.67 (dd, *J* = 16.4, 8.0 Hz, 1H); ^13^C NMR (75 MHz, DMSO-*d*_6_): δ 173.2, 172.3, 166.0, 153.7, 151.3, 149.3, 147.9, 143.4, 138.4, 128.8, 128.7, 127.9, 127.4, 127.1, 117.5, 102.9, 49.7, 43.8, 36.6, 31.3, 29.9; MS (ES^–^) *m*/*z* calcd for C_25_H_24_N_6_O_7_: 520.2; found: 519.0 [M – H]^−;^ HPLC *t*_R_ = 15.48 min, 97.4%.

#### *N*-(4-{[1-(3,4-Dichlorobenzyl)-3,7-dimethyl-2,6-dioxo-2,3,6,7-tetrahydro-1*H*-purin-8-yl]amino}benzoyl)-l-aspartic Acid (**12**)

^1^H NMR (300 MHz, DMSO-*d*_6_): δ 12.70 (bs, 2H), 9.48 (s, 1H), 8.53 (d, *J* = 8.1 Hz, 1H), 7.82 (d, *J* = 8.7 Hz, 2H), 7.65 (d, *J* = 9.0 Hz, 2H), 7.55 (d, *J* = 8.4 Hz, 1H), 7.54 (s, 1H), 7.27 (dd, *J* = 8.4, 2.4 Hz, 1H), 5.01 (s, 2H), 4.71–4.68 (m, 1H), 3.79 (s, 3H), 3.41 (s, 3H), 2.81 (dd, *J* = 16.8, 6.3 Hz, 1H), 2.66 (dd, *J* = 16.5, 7.8 Hz, 1H); ^13^C NMR (75 MHz, DMSO-*d*_6_): δ 173.4, 172.5, 165.9, 153.6, 151.3, 149.4, 148.1, 143.3, 139.6, 131.3, 130.9, 130.1, 128.8, 128.4, 127.3, 117.5, 102.9, 49.7, 42.9, 37.2, 31.2, 29.9; MS (ES^–^) *m*/*z* calcd for C_25_H_22_Cl_2_N_6_O_7_: 588.1; found: 587.0 [M – H]^−^; HRMS (ESI^–^) calcd for C_25_H_21_Cl_2_N_6_O_7_: 587.0848; found: 587.0843 [M – H]^−^; HPLC *t*_R_ = 18.92 min, 96.3%.

#### *N*-[4-({1-[(4-Chloro-1*H*-indol-2-yl)methyl]-3,7-dimethyl-2,6-dioxo-2,3,6,7-tetrahydro-1*H*-purin-8-yl}amino)benzoyl]-l-glutamic Acid (**13**)

^1^H NMR (400 MHz, DMSO-*d*_6_): δ 11.27 (s, 1H), 9.46 (s, 1H), 8.41 (d, *J* = 7.6 Hz, 1H), 7.86 (d, *J* = 9.2 Hz, 2H), 7.75 (d, *J* = 8.8 Hz, 2H), 7.32 (d, *J* = 8.4 Hz, 1H), 7.03–6.97 (m, 2H), 6.25 (s, 1H), 5.19 (s, 2H), 4.39–4.30 (m, 1H), 3.82 (s, 3H), 3.45 (s, 3H), 2.34 (t, *J* = 7.4 Hz, 2H), 2.08–2.04 (m, 1H), 1.96–1.91 (m, 1H); ^13^C NMR (75 MHz, DMSO-*d*_6_): δ 174.3, 174.0, 166.5, 153.5, 151.3, 149.4, 148.1, 143.3, 137.4, 137.0, 128.9, 127.2, 126.7, 124.0, 122.0, 118.9, 117.5, 110.9, 102.9, 98.1, 52.3, 37.9, 31.3, 30.9, 30.0, 26.4; MS (ES^–^) *m*/*z* calcd for C_28_H_26_ClN_7_O_7_: 607.2; found: 606.1 [M – H]^−^; HRMS (ESI^–^) calcd for C_28_H_25_ClN_7_O_7_: 606.1504; found: 606.1496 [M – H]^−^; HPLC *t*_R_ = 18.39 min, 98.1%.

#### *N*-{4-[(1-Benzyl-3,7-dimethyl-2,6-dioxo-2,3,6,7-tetrahydro-1*H*-purin-8-yl)amino]benzoyl}-l-glutamic Acid (**14**)

^1^H NMR (400 MHz, DMSO-*d*_6_): δ 12.40 (bs, 2H), 9.45 (s, 1H), 8.40 (d, *J* = 6.4 Hz, 1H), 7.85 (d, *J* = 8.0 Hz, 2H), 7.75 (d, *J* = 8.8 Hz, 2H), 7.29–7.27 (m, 4H), 7.22 (s, 1H), 5.04 (s, 2H), 4.38–4.36 (m, 1H), 3.80 (s, 3H), 3.42 (s, 3H), 2.35–2.33 (m, 2H), 2.07–2.05 (m, 1H), 1.94–1.92 (m, 1H); ^13^C NMR (75 MHz, DMSO-*d*_6_): δ 174.3, 174.1, 166.5, 153.9, 151.3, 149.3, 148.2, 143.3, 138.9, 129.2, 128.7, 127.9, 127.4, 127.2, 117.5, 102.9, 53.2, 43.8, 31.2, 30.9, 29.9, 25.9; MS (ES^–^) *m*/*z* calcd for C_26_H_26_N_6_O_7_: 534.2; found: 533.1 [M – H]^−^; HRMS (ESI^–^) calcd for C_26_H_25_N_6_O_7_: 533.1784; found: 533.1764 [M – H]^−^; HPLC *t*_R_ = 15.65 min, 99.9%.

#### *N*-(4-{[1-(3,4-Dichlorobenzyl)-3,7-dimethyl-2,6-dioxo-2,3,6,7-tetrahydro-1*H*-purin-8-yl]amino}benzoyl)-l-glutamic Acid (**15**)

^1^H NMR (400 MHz, DMSO-*d*_6_): δ 12.38 (bs, 2H), 9.47 (s, 1H), 8.42 (d, *J* = 6.4 Hz, 1H), 7.85 (d, *J* = 7.6 Hz, 2H), 7.75 (d, *J* = 7.6 Hz, 2H), 7.55 (d, *J* = 8.4 Hz, 1H), 7.54 (s, 1H), 7.27 (d, *J* = 7.6 Hz, 1H), 5.01 (s, 2H), 4.42–4.32 (m, 1H), 3.79 (s, 3H), 3.41 (s, 3H), 2.42–2.30 (m, 2H), 2.18–2.00 (m, 1H), 2.00–1.82 (m, 1H); ^13^C NMR (75 MHz, DMSO-*d*_6_): δ 174.4, 174.0, 166.5, 153.6, 151.2, 149.4, 148.1, 143.2, 139.6, 131.3, 130.9, 130.1, 128.9, 128.4, 127.2, 117.5, 102.8, 52.3, 42.9, 31.2, 30.9, 29.9, 26.4; MS (ES^–^) *m*/*z* calcd for C_26_H_24_Cl_2_N_6_O_7_: 602.1; found: 601.0 [M – H]^−^; HRMS (ESI^+^) calcd for C_26_H_24_Cl_2_N_6_NaO_7_: 625.0981; found: 625.0981 [M + Na]^+^; HPLC *t*_R_ = 19.12 min, 99.1%.

#### 4-({[1-(3,4-Dichlorobenzyl)-3-methyl-2,6-dioxo-1,2,3,6-tetrahydropyrimidin-4-yl]carbamoyl}amino)benzoic Acid (**17**)

^1^H NMR (400 MHz, DMSO-*d*_6_): δ 12.68 (s, 1H), 12.30 (s, 1H), 10.81 (s, 1H), 8.35 (s, 1H), 7.87 (d, *J* = 8.8 Hz, 2H), 7.67 (d, *J* = 8.4 Hz, 2H), 7.56 (d, *J* = 8.0 Hz, 2H), 7.29 (d, *J* = 8.0 Hz, 1H), 5.01 (s, 2H), 3.34 (s, 3H); ^13^C NMR (75 MHz, DMSO-*d*_6_): δ 167.3, 166.9, 163.2, 158.9, 149.5, 143.0, 138.9, 131.4, 130.9, 130.2, 129.9, 128.3, 125.5, 119.4, 81.2, 43.6, 30.4; MS (ES^–^) *m*/*z* calcd for C_20_H_16_Cl_2_N_4_O_5_: 462.0; found: 461.0 [M – H]^−^; HRMS (ESI^–^) calcd for C_20_H_15_Cl_2_N_4_O_5_: 461.0419; found: 461.0414 [M – H]^−^; HPLC *t*_R_ = 24.28 min, 99.5%.

#### *N*-[4-({[1-(3,4-Dichlorobenzyl)-3-methyl-2,6-dioxo-1,2,3,6-tetrahydropyrimidin-4-yl]carbamoyl}amino)benzoyl]-l-glutamic Acid (**18**)

^1^H NMR (400 MHz, DMSO-*d*_6_): δ 12.23 (s, 1H), 10.84 (s, 1H), 8.49 (d, *J* = 6.4 Hz, 1H), 8.33 (s, 1H), 7.84 (d, *J* = 7.2 Hz, 2H), 7.66 (d, *J* = 7.2 Hz, 2H), 7.57 (s, 2H), 7.29 (d, *J* = 8.0 Hz, 1H), 5.02 (s, 2H), 4.37 (bs, 1H), 3.34 (s, 3H), 2.42–2.26 (m, 2H), 2.18–2.00 (m, 1H), 2.00–1.82 (m, 1H); ^13^C NMR (75 MHz, DMSO-*d*_6_): δ 174.3, 174.0, 166.8, 166.4, 163.2, 158.9, 149.6, 141.8, 138.9, 131.4, 130.9, 130.1, 129.8, 129.0, 128.7, 128.2, 119.3, 81.4, 52.4, 42.7, 30.9, 30.4, 26.4; MS (ES^+^) *m*/*z* calcd for C_25_H_23_Cl_2_N_5_O_8_: 591.1; found: 592.1 [M + H]^+^; HRMS (ESI^–^) calcd for C_25_H_22_Cl_2_N_5_O_8_: 590.0845; found: 590.0849 [M – H]^−^; HPLC *t*_R_ = 19.65 min, 99.4%.

#### *N*-{[3-(3,4-Dichlorobenzyl)-2,4-dioxo-1,2,3,4-tetrahydroquinazolin-6-yl]carbonyl}-l-glutamic Acid (**20**)

^1^H NMR (400 MHz, DMSO-*d*_6_): δ 12.40 (bs, 2H), 11.8 (s, 1H), 8.81 (d, *J* = 7.6 Hz, 1H), 8.55 (s, 1H), 8.13 (d, *J* = 6.8 Hz, 1H), 7.60 (s, 1H), 7.55 (d, *J* = 8.4 Hz, 1H), 7.31 (d, *J* = 6.8 Hz, 1H), 7.24 (d, *J* = 8.0 Hz, 1H), 5.07 (s, 2H), 4.42–4.30 (m, 1H), 2.40–2.11 (m, 2H), 2.10–2.00 (m, 1H), 2.00–1.85 (m, 1H); ^13^C NMR (100 MHz, DMSO-*d*_6_): δ 174.3, 173.8, 165.7, 162.3, 150.6, 142.2, 138.8, 134.8, 131.4, 130.9, 130.2, 130.1, 128.4, 127.6, 115.7, 113.8, 52.6, 42.8, 30.8, 26.3; MS (ES^+^) *m*/*z* calcd for C_21_H_17_Cl_2_N_3_O_7_: 493.0; found: 494.1 [M + H]^+^; HRMS (ESI^–^) calcd for C_21_H_16_Cl_2_N_3_O_7_: 492.0365; found: 492.0403 [M – H]^−^; HPLC *t*_R_ = 16.73 min, 91.9%.

### Protein Expression and Purification

The cDNA fragment encoding human MTHFD2 residues 36–350 was cloned into pET14b (Novagen, Madison) attached with a N-terminal 6×-histidine tag followed by a thrombin cleavage site. It was overexpressed 6 h after induction in the bacterial strain BL21(DE3). Pellets were harvested and frozen at −80 °C, then lysed by sonication in lysis buffer (50 mM Tris, pH 7.8, 250 mM NaCl). MTHFD2 proteins were purified with a HisTrap-HP column (Cytiva, Marlborough). After washing, the eluant containing MTHFD2 proteins was exchanged with thrombin cleavage buffer (20 mM Tris, pH 8.2, 150 mM NaCl, 2.5 mM CaCl_2_) using a HiPrep 26/10 Desalting column (Cytiva, Marlborough) and digested with the thrombin protease overnight at 4 °C. After thrombin digestion, a final concentration of 1 mM TCEP was added, and the MTHFD2 protein solution was concentrated to 7 mg/mL.

### Crystallization and the Soaking Experiment

Crystals of MTHFD2/**15**/**21**, MTHFD2/**9**/**21**, and MTHFD2/**3**/**21** were prepared by the soaking method. The crystals for the soaking experiment were produced following the similar process as that for MTHFD2/NAD^+^/P_i_/**21** but without NAD^+^, MgCl_2_, and Na_2_HPO_4_ added (see the Supporting Information). The concentration of **21** was decreased to 0.2 mM and the reservoir solution was modified to 23% isopropanol, 0.1 M bis-Tris, pH 7.1, 4% PEG200, 3% PEG3350, and 6% glycerol. After 1 week, the cocrystals were ready for the following soaking experiment. **15**, **9**, or **3** was directly added to the crystallization drop at 2 mM and equilibrated for 30 min to 5 h at 18 °C. Crystals were flash frozen in liquid nitrogen with DMSO added to the drop as a freeze protectant.

### Structure Determination

Diffraction data were collected at the beamline TPS05A (NSRRC, Taiwan) and processed using the software program HKL2000.^34^ The structures were solved by the molecular replacement method MOLREP^35^ of the CCP4^36^ program suites using the MTHFD2 structure (PDB 5TC4)^14^ as an initial search model. Following refinement and model building were processed by utilizing PHENIX^37^ and COOT.^38^ Figures of structures were generated by PyMOL (Schrödinger).

### Enzymatic Assays

Human MTHFD2 and MTHFD1 proteins were purified as described by Gustafsson et al.^14^ For constructing MTHFD2 and MTHFD1-overexpressing plasmids, human MTHFD2 cDNA (amino acids 36–350) and human MTHFD1 cDNA (amino acids 1–306) were generated by PCR from human testis cDNA and cloned to the pBacPAK8-MTGFP-His vector. C-terminal-His-tagged MTHFD2 and MTHFD1 were amplified by the insect cell overexpression system and purified by the HisTrap-HP column (Cytiva, Marlborough).

MTHFD1 and MTHFD2 activity assays were performed and modified as described by Mejia et al.^4^ Purified MTHFD2 was preincubated with 0.4 mM NAD^+^ for 10 min and MTHFD1 was incubated with 2 mM NADP^+^. Then, these enzyme/cofactor mixtures were incubated with compounds for a further 10 min. The enzymatic reaction was initiated by adding MTHFD2 assay buffer (30 mM potassium phosphate, pH 7.3, 0.15 mM tetrahydrofolate, 2.5 mM formaldehyde, 6 mM MgCl_2_) or MTHFD1 assay buffer (30 mM potassium phosphate, pH 7.3, 0.3 mM tetrahydrofolate, 2.5 mM formaldehyde, 6 mM MgCl_2_) and incubated for 10 min. The reaction was stopped by addition of HCl (final 0.18 N) and the absorbance at 350 nm was determined.

## Supporting Information Available

Crystallization of MTHFD2/NAD+/P_i_/**21**; chemical structure of compound **21**; HPLC trace and ^1^H NMR spectrum of **15** (PDF)
Molecular formula strings (CSV)

## Accession Codes

7EHJ (MTHFD2/NAD^+^/P_i_/**21**); 7EHM (MTHFD2/**15**/**21**); 7EHN (MTHFD2/**9**/**21**); 7EHV (MTHFD2/**3**/**21**). The authors will release the atomic coordinates and experimental data upon article publication.

## Abbreviations

MTHFD2: methylenetetrahydrofolate dehydrogenase 2
SAR: structure–activity relationships
