# Supplementary material for: Xanthine Derivatives Reveal an Allosteric Binding Site in Methylenetetrahydrofolate Dehydrogenase 2 (MTHFD2)
Source: J Med Chem. 2021 Aug 2;64(15):11288–301. doi: 10.1021/acs.jmedchem.1c00663 (PMC8389891; doi:10.1021/acs.jmedchem.1c00663)
Supplement: Supplementary file 1 — jm1c00663_si_001.pdf [file jm1c00663_si_001.pdf]

Supporting Information

Xanthine Derivatives Reveal an Allosteric Binding Site in Methylenetetrahydrofolate Dehydrogenase 2 (MTHFD2)

Lung-Chun Lee,[†] *Yi-Hui Peng,* [†#] *Hsin-Huei Chang,* [†#]*Tsu-Hsu,* [†#] *Cheng-Tai Lu,*[†] *Chih-Hsiang Huang,*[†] *Ching-Cheng Hsueh,*[†] *Fang-Chun Kung,*[†] *Ching-Chuan Kuo,*[†§*] *Weir-Torn Jiaang,* [†*] *and Su-Ying Wu*[†*]

[†] Institute of Biotechnology and Pharmaceutical Research, National Health Research Institutes, 35, Keyan Road, Zhunan Town, Miaoli County 350, Taiwan, R.O.C.

[§]Graduate Institute of Biomedical Sciences, China Medical University, Taichung 406, Taiwan, R.O.C.

[#] Yi-Hui Peng, Hsin-Huei Chang and Tsu-Hsu contributed equally

Corresponding author:

Su-Ying Wu, Email:suying@nhri.org.tw

Weir-Torn Jiaang, Email:wtjiaang@nhri.org.tw

Ching-Chuan Kuo, Email:cckuo@nhri.org.tw

Table of Contents

Crystallization of MTHFD2/NAD$^+$/P_i/21 .... S3
Figure S-1. Chemical structure of compound 21 .... S3
Figure S-2. HPLC trace and ^{1}H NMR spectrum of 15 .... S4
Reference S5

Crystallization of MTHFD2/NAD$^+$/P$_i$/21

Co-crystals of human MTHFD2, NAD$^+$, phosphate and a weak inhibitor, (4-(3-(4-amino-6-hydroxypyrimidin-5-yl)ureido)benzoyl)-L-glutamic acid (**21**, IC_{50} = 8.33 μM in the MTHFD2 enzymatic assay) were prepared by hanging-drop vapor diffusion method. **21** can be prepared according to previously described methods.[1] 2 mM **21**, 5 mM NAD$^+$ and 6 mM $MgCl_2$ were added to purified human MTHFD2 protein and incubated on ice for 40 min. Na_2HPO_4 was added to a final concentration of 10 mM and equilibrated for 10 min. The mixture of protein and **21** was 1:1 (v/v) mixed with reservoir solution (12% isopropanol, 0.1 M bis-Tris, pH 6.5, 3% PEG200) and incubated at 18 ºC. After 1 week, the crystals were flash-frozen in liquid nitrogen with DMSO added to the drop as a freeze protectant and subjected for data collection.

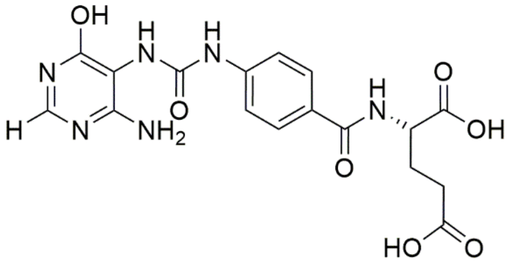

21

Figure S-1. Chemical structure of **21**.

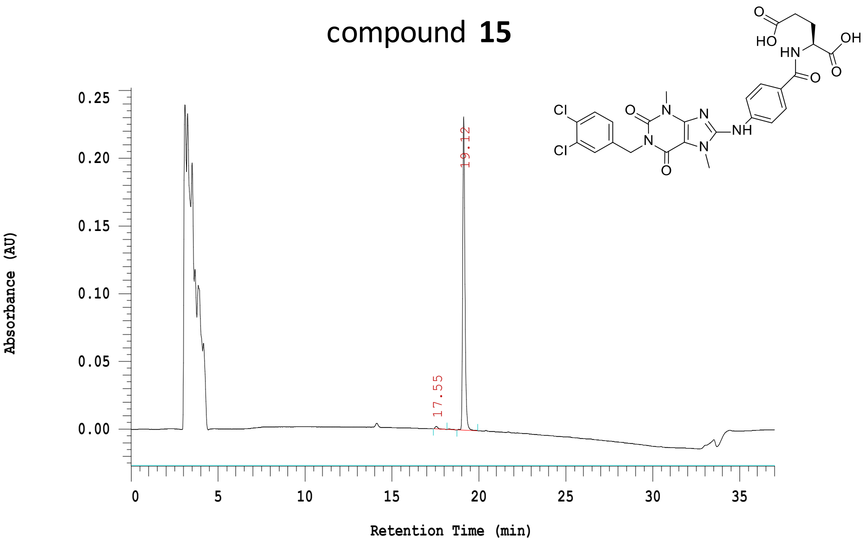

Peak Quantitation: AREA
Calculation Method: AREA%

No.	RT	Area	Height	Conc 1
1	17.55	9560	943	0.941
2	19.12	1005861	115465	99.059
		1015421	116408	100.000

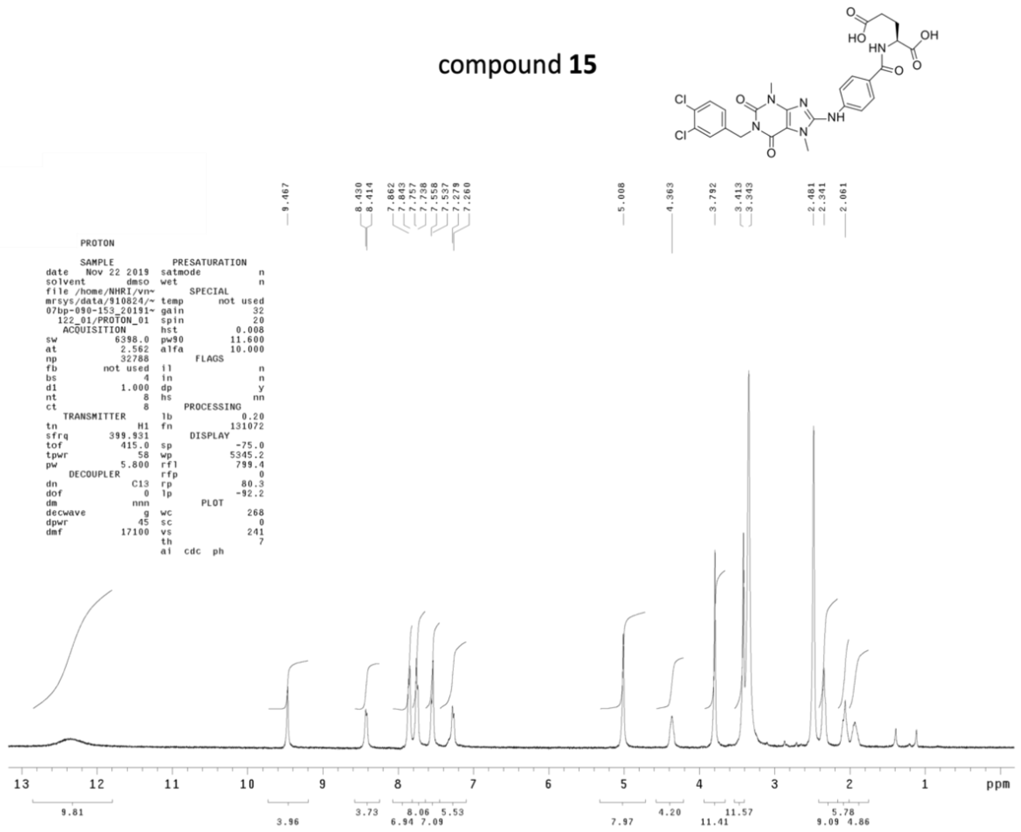

Figure S-2. HPLC trace and ^{1}H NMR spectrum of **15**.

Reference

1. Eadsforth, T. C.; Pinto, A.; Luciani, R.; Tamborini, L.; Cullia, G.; De Micheli, C.; Marinelli, L.; Cosconati, S.; Novellino, E.; Presti, L. L.; Cordeiro da Silva, A.; Conti, P.; Hunter, W. N.; Costi, M. P. Characterization of 2,4-diamino-6-oxo-1,6-dihydropyrimidin-5-yl ureido based inhibitors of trypanosoma brucei FolD and testing for antiparasitic activity. *J. Med. Chem.* **2015**, *58*, 7938−7948.
